# Factors Associated with Early Mortality in Acute Type A Aortic Dissection—A Single-Centre Experience

**DOI:** 10.3390/jcm13041023

**Published:** 2024-02-10

**Authors:** Panagiotis Doukas, Nicola Dalibor, András Keszei, Jelle Frankort, Julia Krabbe, Rachad Zayat, Michael J. Jacobs, Alexander Gombert, Payam Akhyari, Arash Mehdiani

**Affiliations:** 1 European Vascular Center Aachen-Maastricht, Department of Vascular Surgery, RWTH University Hospital Aachen, Pauwelsstraße 30, 52074 Aachen, Germany; nicola.dalibor@rwth-aachen.de (N.D.); jefrankort@ukaachen.de (J.F.); mjacobs@ukaachen.de (M.J.J.); agombert@ukaachen.de (A.G.); 2 Center for Translational & Clinical Research Aachen (CTC-A), RWTH Aachen University, Pauwelsstraße 30, 52074 Aachen, Germany; akeszei@ukaachen.de; 3 Institute of Occupational, Social and Environmental Medicine, Medical Faculty, RWTH Aachen University, Pauwelsstraße 30, 52074 Aachen, Germany; 4 Clinic for Cardiac Surgery, University Hospital RWTH Aachen, Pauwelsstraße 30, 52074 Aachen, Germany; rzayat@ukaachen.de (R.Z.); pakhyari@ukaachen.de (P.A.); amehdiani@ukaachen.de (A.M.)

**Keywords:** aortic dissection, type A, management, outcomes, complications

## Abstract

Background: Acute aortic dissection type A (AADA) is a surgical emergency with relevant mortality and morbidity despite improvements in current management protocols. Identifying patients at risk of a fatal outcome and controlling the factors associated with mortality remain of paramount importance. Methods: In this retrospective observational study, we reviewed the medical records of 117 patients with AADA, who were referred to our centre and operated on between 2005 and 2021. Preoperative, intraoperative, and postoperative variables were analysed and tested for their correlation with in-hospital mortality. Results: The overall survival rate was 83%. Preoperatively, factors associated with mortality were age (*p* = 0.02), chronic hypertension (*p* = 0.02), any grade of aortic valve stenosis in the patient’s medical history (*p* = 0.03), atrial fibrillation (*p* = 0.04), and oral anticoagulation (*p* = 0.04). Non-survivors had significantly longer operative times (*p* = 0.002). During the postoperative phase, mortality was strongly associated with acute kidney injury (AKI) (*p* < 0.001), acute heart failure (*p* < 0.001), stroke (*p* = 0.02), focal neurological deficits (*p* = 0.02), and sepsis (*p* = 0.001). In the multivariate regression analysis, the onset of postoperative focal neurological deficits was the best predictor of a fatal outcome after adjusting for ARDS (odds ratio: 5.8, 95%-CI: 1.2–41.7, *p* = 0.04). Conclusions: In this retrospective analysis, atrial fibrillation, oral anticoagulation, hypertension, and age were significantly correlated with mortality. Postoperatively, acute kidney injury, acute heart failure, sepsis, and focal neurological deficits were correlated with in-hospital mortality, and focal neurological deficit has been identified as a significant predictor of fatal outcomes. Early detection and interdisciplinary management of at-risk patients remain crucial throughout the postoperative phase.

## 1. Introduction

Aortic dissection involving the ascending aorta and/or the aortic arch, defined as type A aortic dissection according to the Stanford classification, remains an acute, critical pathology requiring emergent surgical treatment. Although diagnostic and treatment protocols have been continuously improved and optimized through the years, the associated mortality rates are still significant (17.7% to 22% in-hospital mortality and a 65% survival rate over the following 10 years) [1,2]. Many aspects of the surgical management of acute aortic dissections type A (AADA), such as the extent of distal aortic reconstruction and management of the aortic root, among others, remain the subject of debate among clinicians [3], since reducing the complexity of surgery may improve early outcomes on the one hand, but may lead to increased risk of reoperation on the other [4]. 

Furthermore, complications in the postoperative phase after this challenging procedure may lead to adverse events, potentially affecting the patients’ prognosis and survival [5]. Prompt identification of patients at risk for complications and early intervention may improve outcomes and reduce in-hospital mortality. 

In this retrospective observational study, we report our experience with AADA cases at our centre over the last 16 years. This study aimed to identify the factors that may influence the postoperative prognosis. 

## 2. Materials and Methods

### 2.1. Patients

In this study, we retrospectively reviewed the medical records of 117 patients with acute AADA, who were referred to our centre and underwent surgery between 2005 and 2021. The exclusion criteria were subacute type A aortic dissection, defined as older than 2 weeks; chronic aortic dissection, defined as older than 90 days [6]; patients declared dead on arrival in the emergency department; and patients that did not undergo surgery. The study was reviewed and approved by the ethics committee of the University Hospital RWTH Aachen (EK 20-003) and was designed according to the STROBE criteria and the Declaration of Helsinki. 

### 2.2. Surgery

After the initial clinical evaluation in the emergency department and radiologic confirmation of diagnosis through an ECG-gated CT scan, the patients were brought to the operating room. The operating team consisted of two experienced surgeons and a senior resident. The surgical protocol was initiated with median sternotomy and establishment of cardiopulmonary bypass (CBP) under full heparinization. The right subclavian, right axillary, or left femoral artery was used as an access site for arterial cannulation. In some cases, direct cannulation of the aorta was possible. A two-stage venous cannula was placed in most cases in the right atrium, with femoral venous cannulation as a second option, according to the standard procedure as described in the literature [7]. Cerebral perfusion was established unilaterally or bilaterally in an antegrade manner. During the early phase of the investigated period, patients with pathologies limited to the ascending aorta did not undergo selective cerebral perfusion. After initiation of the CBP, depending on the extent of the planned distal reconstruction, the procedure was performed under hypothermic circulatory arrest and the patient was cooled down to 18 °C. Antegrade cardioplegia with Bretschneider cardioplegia solution (Custodiol^®^, Dr. F. Köhler Chemie GmbH., Bensheim, Germany) was used to protect the myocardium. Aortic root management was dependent on valve functionality and on whether the aortic root was affected by the dissection. If possible, the aortic root was managed conservatively or with valve-sparing reconstruction using the David technique. The extent of distal repair was decided depending on the state of the aortic arch and descending aorta. Total arch repair was performed in cases of complex tears in the aortic arch, aneurysmatic expansion, or rupture of the arch. The supra-aortic vessels were reimplanted individually or using the island technique. In cases of extensive pathologies involving the descending aorta, the frozen elephant trunk technique was performed. After completion of the distal part of the reconstruction, the air was removed from the graft, antegrade perfusion was resumed, and systemic rewarming was initiated. The period from the removal of the aortic clamp until systemic rewarming is complete is subsequently referred to as the “reperfusion time”. Cerebral perfusion was monitored throughout the procedure using near-infrared spectroscopy (NIRS) [8,9]. 

### 2.3. Definitions

If the sequential organ failure assessment score (SOFA) was increased by two or more points from the previous day and clinical suspicion of infection was in place, the diagnosis of sepsis was confirmed [10]. Compromised function of the liver (spontaneous international normalized ratio of >1.5) and acute jaundice satisfied the criteria for the diagnosis of acute liver failure [11]. Acute kidney injury (AKI) was diagnosed according to the Kidney 100 Disease Improving Global Outcomes (KDIGO) criteria [12]. Acute heart failure was diagnosed in patients with the clinical presentation of cardiogenic shock, pulmonary oedema, or congestive heart failure [13]. Shock was defined as having a systolic blood pressure below 90 mmHg or necessitating mechanical or pressor support to uphold values above this threshold [14]. Spinal ischaemia was assessed with the modified Tarlov scale [15]. Postoperative ischaemic strokes were detected using either CT or MRI scans [16], and focal neurological deficits were assessed through clinical examination by a neurologist. The gastrointestinal complications reported in this study summarize cases of gastrointestinal bleeding and/or mesenteric ischaemia [17]. The diagnosis of acute respiratory distress syndrome (ARDS) and its classification were made based on the Berlin criteria [18]. Failure of two or more vital organ systems fulfilled the criteria for the diagnosis of multiple organ dysfunction syndrome (MODS) [19]. Dissection-related obstruction of the aortic branches and the following hypoperfusion of the end-organ were defined as malperfusion [20]. Gastrointestinal complications ranged from gastrointestinal bleeds to transmural intestinal necrosis [21]. 

### 2.4. Statistics

Continuous variables were described using the mean and standard deviation. Categorical variables were tabulated using frequencies and percentages. The occurrence of death was modelled using logistic regression models. Predefined sets of independent variables were used in the models. Missing observations of categorical variables were modelled as separate categories. Missing continuous variables were imputed using multiple imputations with chained equations using predictive mean matching. Fifty imputed datasets were generated, and Rubin’s rule was used to pool the parameter estimates. Data analysis was performed using the R language and environment for statistical computing (Version 4.1.0 R Foundation for Statistical Computing, Vienna, Austria. URL (accessed on 11 November 2023) https://www.R-project.org/). Best subset model selection was performed using the glmulti package (Version 1.0.8) [22], and the mice package (Version 3.14.0) [23] was used for imputations.

## 3. Results

### 3.1. Demographics and Clinical Presentation

**Figure 1:**
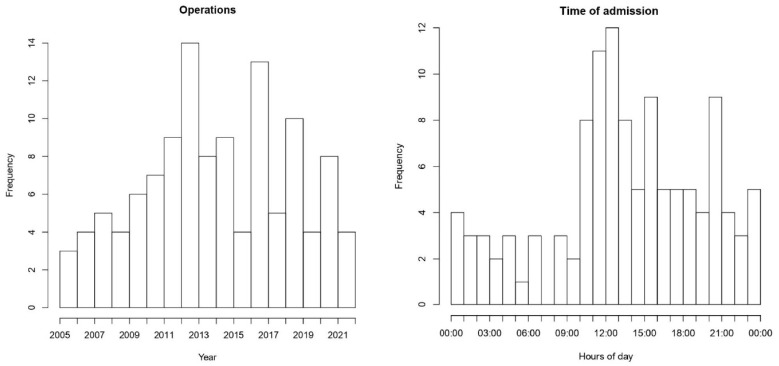
Frequency of operations per year and time of patient admission.

Between March 2005 and October 2021, on average, 7.3 patients per year were operated on (Figure 1). Most patients were admitted at noon (Figure 1). The median duration from hospital admission to surgery was 91 min [IQR 69-145]. The majority of patients (50.3%) were directly transported to the emergency room of our centre after an ambulance was alerted about their symptoms, while 52 patients (44.4%) were transferred from other hospitals following diagnosis confirmation. Urgent referrals from primary care institutions and general practitioners accounted for five patients (4.2%). Transportation to the emergency room was predominantly via ambulance for most patients (91.4%), with medical air rescue utilized in ten cases (8.6%). In two instances, treatment was delayed due to the patient’s initial admission to another ward with a different primary diagnosis.

Of the 117 analysed patients, 31 were women (26%). There was a significant difference in age between survivors and non-survivors (58.4 ± 11.4 vs. 65.3 ± 11; *p* = 0.02). Patients on anticoagulants (*p* = 0.04), those with chronic atrial fibrillation (*p* = 0.04), and those with chronic hypertension (*p* = 0.02) had a significantly increased risk of fatal outcomes. We also observed a positive association between mortality and aortic valve stenosis (*p* = 0.03). The clinical presentation of spinal ischaemia varied across the cohort and ranged from mild paraparesis in seven patients (modified Tarlov scale score of four) to complete paraplegia in four patients (modified Tarlov scale score of zero). Additionally, five patients presented a moderate form of paraplegia (modified Tarlov score of two). The details of the patient demographics and clinical presentations at admission are summarized in Table 1. Using the classification system proposed by the Society for Vascular Surgery (SVS) and the Society of Thoracic Surgeons (STS) [24,25], entry tears were found in the vast majority of cases in Zone 0. In one case, the tear was in Zone 1, and in two cases, the entries were found in Zone 2 or further below in the descending aorta and progressed retrogradely to involve the ascending aorta. In most cases, dissection progressed to the aortoiliac bifurcation. We did not find any association between the localization of the entry tear or extent of dissection and mortality. Cerebral and renovisceral malperfusion—as revealed by CT scans [26]—were also not significantly correlated with in-hospital mortality (Supplementary Table S1). 

### 3.2. Operative Details

In the total of the present cohort, aortic reconstruction was limited to the ascending aorta in 35 patients (30%). Hemiarch replacement was performed in 28 cases (24%), extensive distal reconstruction with total arch replacement was performed in 5 cases (4%), and the frozen elephant trunk technique was performed in 49 cases (42%). If simultaneous valve repair was necessary, biological valve conduits were the most common, with 29 cases (25%), followed by mechanical Bentall repairs (28 cases, 24%). Nine patients (8%) underwent valve-sparing aortic root repair. There was no significant correlation between different operative modalities and patient mortality (Table 2). We observed significantly longer operation times in the non-survivor group (minutes: 364.6 ± 97.1 vs. 449.1 ± 141.7, *p* = 0.002). Nineteen patients required operative revision for bleeding, a complication observed in 25% of the cases with fatal outcomes. The origin of the bleeding remained elusive in seven cases, while in nine cases, it presented as diffuse. Additionally, in two cases, the bleeding emanated directly from the right ventricle, and in one case, it originated from the proximal anastomosis.

### 3.3. Postoperative Phase

**Figure 2:**
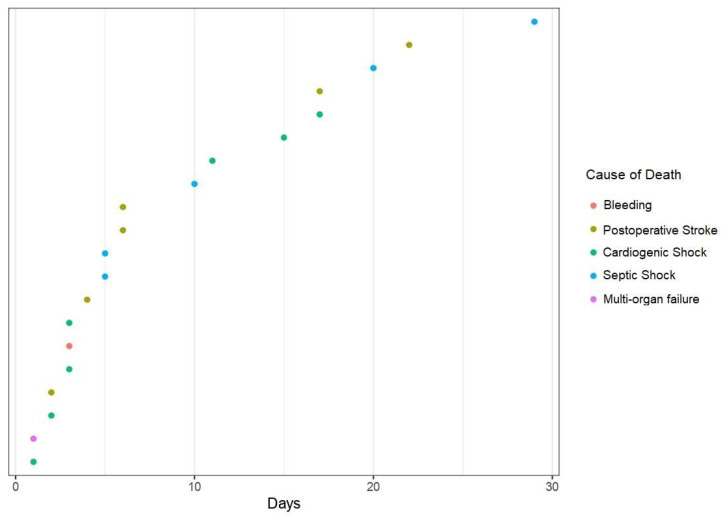
Causes and time points of fatal outcomes.

Twenty patients died in hospital. Cardiogenic shock and postoperative stroke were the most common causes of death in our cohort, accounting for 60% of the fatal outcomes, followed by septic shock in five cases (Table 3). The time points of the registered deaths, along with their respective causalities, are shown in Figure 2.

AKI and acute heart failure (*p* < 0.001 for both), sepsis (*p* = 0.04), and focal neurological deficits (*p* = 0.02) were strongly associated with in-hospital mortality. ARDS of all stages was more common in the non-survivor group (24% vs. 6%; *p* = 0.06). Acute limb ischaemia was found to be strongly associated with poor prognosis (18% vs. 3%; *p* = 0.06). Details of the postoperative complications and their association with mortality are presented in Table 3.

Clinical factors associated with poor outcomes and end-organ damage were included in a univariate regression model (Supplementary Table S2). After evaluating every possible subset combination, the best fitting model of mortality included the onset of focal neurological deficits and ARDS (Supplementary Table S3). Patients with focal neurological deficits had an odds ratio of 5.8 (95% confidence interval: 1.2–41.7; *p* = 0.04) for mortality adjusted for ARDS.

## 4. Discussion

Despite modern advancements in its detection and treatment, AADA remains a challenging aortic pathology, with relevant mortality rates. A recent study investigating the epidemiologic characteristics of AADA in the Danish population revealed an incidence rate of 2.2/100.000 and a 30-day mortality rate of 22% [27], confirming the rather uncommon but fatal nature of the disease. The risk factors usually associated with AADA are hypertension, atherosclerosis, aneurysmatic degeneration of the aorta, and connective tissue disease [2,28]. In this retrospective, observational study, we examined the pre- and perioperative parameters of patients admitted with AADA in our centre, as well as their postoperative course and complications, in order to elucidate the correlations of the parameters associated with poor outcomes and identify the patients at risk. 

Regarding the preoperative characteristics of our patient cohort, hypertension was the most common comorbidity (64% of all patients), and it was significantly correlated with mortality (*p* = 0.02). This observation is in line with the findings of Wang et al., who described hypertension as an independent risk factor for long-term modality [29]. Another factor associated with early mortality is atrial fibrillation [30]. Patients with atrial fibrillation and acute aortic syndrome reportedly have a higher in-hospital mortality than do those without atrial fibrillation [30]. These patients are often on oral anticoagulation, which has also been described as a risk factor for bleeding and haemodynamic instability after AADA surgery [31]. In line with these findings, our results indicated both atrial fibrillation (*p* = 0.04) and oral anticoagulation (*p* = 0.04) to be strongly associated with mortality. Postoperative bleeding was observed in 25% of the non-survivors in our cohort and was a relevant complication in 19 patients requiring surgical revision. 

The duration of extracorporeal circulation correlates with patients’ survival [5,32]. Survivors in our study showed a trend for shorter reperfusion times (minutes: 83.3 ± 40.6 vs. 98.4 ± 40.5; *p* = 0.25) and total CBP times (minutes: 215.8 ± 73.6 vs. 256.2 ± 125.9; *p* = 0.26) with a significantly shorter total operation time (minutes: 364.6 ± 97.1 vs. 449.1 ± 141.7; *p* = 0.002). Longer applications of extracorporeal circulation reflect the severity of the dissection on the one hand and the patients’ cardiovascular capacity to negotiate reperfusion on the other. Moreover, CBP disrupts the integrity of red blood cells, causing haemolysis; in this way, it may affect microcirculation and organ perfusion [33]. To improve patient outcomes, understanding and optimizing the underlying mechanisms of extracorporeal circulation remain of paramount importance. Simplifying the operation technique might influence the patient’s outcome; in particular, the indication for patients treated with FET should be well justified. In the new era of acute aortic surgery, the application of FET in case of AADA without an entry in the aortic arch is a matter of ongoing discussion.

Organ dysfunction during the postoperative phase may severely affect patient recovery and is associated with adverse outcomes. In our cohort, cardiogenic shock was the most common cause of death, accounting for 30% of all fatal outcomes. In all, 45% of non-survivors were diagnosed with acute heart failure, which was strongly associated with mortality (*p* < 0.001). AKI and a need for renal replacement therapy were also strongly associated with mortality in our cohort (*p* < 0.001), confirming the observations of Huo et al. [5]. AKI after aortic surgery is potentially limiting in terms of patients’ prognosis [34,35,36,37]. In line with this, patients with preoperatively impaired renal function [38] or those with renal malperfusion [39] carry a higher risk of postoperative renal failure. Furthermore, based on the results of the current cohort, chronic kidney disease was not a relevant factor for mortality, and both survivors and non-survivors had comparable serum creatinine levels at admission (survivors: 1.2 ± 1.2 mg/dL; non-survivors: 1 ± 0.4 mg/dL). These findings underline the importance of early detection of postoperative AKI [40] and undeferred initiation of nephroprotective measures to contain damage to the kidney tissue and allow for a quick organ recovery. 

Persistent focal neurological deficits and postoperative stroke were also significantly associated with mortality (*p* = 0.02 for both). Multivariate regression analysis revealed an odds ratio of 5.8 for a fatal outcome in cases of focal neurological deficits. Although the interpretation of this analysis is limited due to the small sample size of the presented cohort, it is in line with clinical observations in the literature. Neurological deficits, with or without evidence of stroke, complicate the patient’s recovery and are associated with high mortality and hospitalization [16], particularly in the elderly [41]. Although intraoperative protocols have been adjusted to monitor and contain cerebral malperfusion, through tedious blood pressure management and intraoperative NIRS monitoring, postoperative neurological complications remain a significant cause of morbidity and mortality [42]. Procedures involving the aortic arch display increased stroke rates [43], and the different arterial cannulation sites have not been found to bring certain benefits in stroke prevention [44]. Identifying and treating affected patients early in the postoperative phase with an interdisciplinary team may improve the overall outcome during the hospital stay and after the patient’s discharge. 

This study is subject to several limitations inherent in retrospective, non-randomized research. The data extraction process was confined to the information available in medical records, which inherently imposes constraints on the depth and comprehensiveness of the dataset. Given that AADA is an emergent pathology, the documentation of patients’ medical history upon admission, and, to a lesser extent, the recording of intraoperative parameters, is frequently found to be insufficient and incomplete. To mitigate potential biases arising from missing data, our statistical analysis considered the absence of observations for categorical variables. Additionally, we employed imputation techniques to address missing values in continuous variables, thereby enhancing the robustness and reliability of our findings. The interpretation of the analysis results should take into account the additional limitation posed by the small sample size within the analysed cohort. Moreover, some of the discoveries presented in this report echo those found in prior publications. Nevertheless, the current study distinguishes itself by providing a comprehensive overview of perioperative predictors of mortality and emphasizes the significance of early postoperative assessment and the screening of end-organ damage, offering a nuanced and detailed exploration of these crucial aspects. Furthermore, the absence of long-term follow-up data for a considerable number of patients post-discharge limits our ability to provide an extended analysis of outcomes. Future trials with robust follow-up mechanisms are warranted to address this limitation and contribute to a more thorough understanding of the extended implications of the surgical modalities for the treatment of AADA.

## 5. Conclusions

Despite improvements in the surgical management of AADA, it still holds relevant mortality and morbidity rates. In this retrospective analysis, we found that atrial fibrillation, oral anticoagulation, and hypertension were significantly correlated with mortality. Postoperatively, acute kidney injury, acute heart failure, and persistent focal neurological deficits were identified as significant predictors of fatal outcomes. Early detection and interdisciplinary management of the patients at risk remain crucial throughout the postoperative phase. 

## Figures and Tables

**Table 1:** Patient demographics and clinical presentation.

	Survivors n = 97 (%)	Non-Survivors n = 20 (%)	Analysed Cases (n)	Association with Mortality (*p*-Value)
**Demographics**				
Age (years)	58.4 ± 11.4	65.3 ± 11	117	0.02 *
Women	28 (29)	3 (15)	117	0.31
BMI (kg/m^2^)	26.9 ± 5.3	27.6 ± 3.3	117	0.52
Smoking	32 (53)	8 (73)	117	0.38
Hypertension	57 (53)	18 (73)	117	0.02 *
Chronic obstructive pulmonary disease	3 (3)	2 (10)	117	0.14
Chronic limb ischaemia	1 (1)	1(5)	117	0.78
Chronic kidney disease	6 (6)	2 (10)	117	0.92
Creatinine, preoperative (mg/dL)	1.2 ± 1.2	1 ± 0.4		0.25
Connective tissue disease	6 (6)	0 (0)	117	0.54
Atrial fibrillation	6 (6)	4 (20)	117	0.04 *
Heart or vascular implants	5 (5)	0 (0)	117	0.65
Heart failure	0 (0)	0 (0)	117	NA
Aortic valve insufficiency in history	4 (4)	2 (10)	117	0.63
Aortic valve stenosis in history	0 (0)	2 (10)	117	0.03 *
Prior CABG	0 (0)	0 (0)	117	NA
Prior valve reconstruction	1 (1)	0 (0)	117	1
**Medication at Admission**				
Beta blockers	9 (23)	1 (17)	45	1
ACE inhibitors	9 (22)	1 (17)	46	1
ARBs	3 (7)	1 (14)	48	1
Acetylsalicylic acid	4 (10)	0 (0)	47	0.88
Oral anticoagulants	6 (6)	4 (20)	117	0.04 *
**Clinical Presentation**				
Thoracic pain	76 (88)	15 (94)	102	0.84
Cardiac arrest	4 (4)	0 (0)	116	0.83
Pericardial effusion	38 (48)	9 (53)	97	0.23
Aortic regurgitation	46 (55)	7 (44)	99	0.56
Unconscious at admission	17 (20)	3 (19)	100	1
Spinal ischaemia	14 (16)	3 (17)	105	1
Acute limb ischaemia	17 (20)	7 (41)	104	0.1
Acute kidney injury	0 (0)	0 (0)	106	NA
Shock	26 (36)	8 (67)	84	0.09

BMI: body mass index; CABG: coronary artery bypass grafting; ACE inhibitor: angiotensin-converting enzyme inhibitors; ARBs: angiotensin II receptor blockers. Statistical significance for *p*-values < 0.05 is marked with *. NA: not applicable

**Table 2:** Operative details.

	Survivors n = 97 (%)	Non-Survivors n = 20 (%)	Analysed Cases (n)	Association with Mortality (*p*-Value)
Duration of operation	364.6 ± 97.1	449.1 ± 141.7	117	0.002 *
CBP temperature (°C)	19 ± 2.4	19.6 ± 2.7	112	0.37
Aortic cross clamp time (minutes)	123.8 ± 46	135.1 ± 72.1	85	0.58
Circulatory arrest time (minutes)	44.2 ± 22.1	53.4 ± 23.5	97	0.18
Reperfusion time (minutes)	83.3 ± 40.6	98.4 ± 40.5	72	0.25
Total bypass time (minutes)	215.8 ± 73.6	256.2 ± 125.9	88	0.26
Arterial canulation site				0.92
Subclavial/Axillary	56 (67)	10 (63)	100	
Femoral	20 (24)	4 (25)		
Aorta	8 (9)	2 (12)		
Venous canulation site			99	0.05
Right atrium	77 (93)	12 (75)		
Femoral	5 (6)	4 (25)		
Bicaval	1 (1)	0 (0)		
Cerebral perfusion			98	0.11
None	19 (23)	1 (6)		
Unilateral	45 (55)	8 (50)		
Bilateral	18 (22)	7 (44)		
**Operative Modalities**				
Ascending aorta repair	29 (30)	6 (30)	117	1
Ascending aorta and hemiarch repair	25 (26)	3 (15)	117	0.46
Ascending aorta and complete arch repair	4 (4)	1 (5)	117	1
Frozen elephant trunk	39 (40)	10 (50)	117	0.65
Conservative root management	46 (47)	13 (65)	117	0.24
Valve-sparing aortic root repair	9 (9)	0 (0)	117	0.34
Bio-Bentall	17 (18)	4 (20)	117	0.96
Mechan. Bentall	25 (26)	3 (15)	117	0.46
Intraoperative CABG	8 (8)	3 (15)	117	0.4
**Postoperative Complications**				
Rethoracotomy: bleeding	14 (14)	5 (25)	117	0.4
Wound infection	6 (6)	0 (0)	117	0.58
N. recurens paralysis	10 (10)	0 (0)	117	0.48

CBP: cardiopulmonary bypass; CABG: coronary artery bypass grafting. Statistical significance for *p*-values < 0.05 is marked with *.

**Table 3:** Postoperative complications.

	Survivors n = 97 (%)	Non-Survivors n = 20 (%)	Analysed Cases (n)	Association with Mortality (*p*-Value)
In-hospital mortality		20 (17)	117	
**Cause of death**			20	
Bleeding		1 (5)		
Postoperative stroke		6 (30)		
Cardiogenic shock		6 (30)		
Septic shock		5 (25)		
Multi-organ failure		1 (5)		
Hospital stay (days)	21.5 ± 13.3	9.5 ± 8	117	0.002 *
ICU stay (days)	12.9 ± 10.6	8.3 ± 8.8	117	0.04 *
Invasive ventilation (hours)	151.1 ± 225.6	156.5 ± 159.5	117	0.9
AKI post-op	31 (32)	16 (80)	117	<0.001 *
RRT	19 (20)	15 (80)	117	<0.001 *
Arrhythmia post-op	48 (49)	10 (50)	117	1
Permanent pacemaker	3 (3)	2 (10)	117	0.43
Atrioventricular block	2 (2)	1 (5)	117	0.44
Aortic valve insufficiency post-op	46 (55)	7 (44)	99	0.56
Acute heart failure	11 (11)	9 (45)	117	<0.001 *
Postoperative stroke	18 (19)	8 (47)	114	0.02 *
Focal neurological deficits	31 (32)	7 (78)	106	0.02 *
Delirium	51 (53)	3 (50)	103	1
Acute limb ischaemia	3 (3)	3 (18)	114	0.06
Pneumonia	54 (56)	16 (64)	112	1
ARDS	7 (7)	4 (27)	112	0.06
Pulmonary artery embolism	2 (2)	0 (0)	114	1
Tracheostomy	24 (25)	3 (27)	99	0.97
GI complications	11 (11)	4 (24)	114	0.32
Liver failure	1 (1)	2 (10)	117	0.13
Sepsis	23 (24)	8 (53)	112	0.04 *

ICU: intensive care unit; AKI: acute kidney injury; RRT: renal replacement therapy; ARDS: acute respiratory distress syndrome; GI: gastrointestinal. Statistical significance for *p*-values < 0.05 is marked with *.

## Data Availability

The data that support the findings of this study are available from the corresponding author, P.D., upon reasonable request.

## Supplementary Materials

The following supporting information can be downloaded at: https://www.mdpi.com/article/10.3390/jcm13041023/s1, Table S1: CT angiography findings on admission. Table S2: Univariate logistic regression model. Table S3: Summary of logistic regression coefficients after best subset selection.
