# Supplementary material for: Factors Associated with Early Mortality in Acute Type A Aortic Dissection—A Single-Centre Experience
Source: J Clin Med. 2024 Feb 10;13(4):1023. doi: 10.3390/jcm13041023 (PMC10889250; doi:10.3390/jcm13041023)
Supplement: Supplementary file 1 [file jcm-13-01023-s001.zip › jcm-2841351-supplementary.pdf]

Supplementary Table S1: CT angiography findings on admission.

	Survivors n=97 (%)	Non-survivors n=20 (%)	Analysed cases (n)	Association with mortality (p-value)
**Entry localization**			91	0.06
0	73 (99)	15 (88)		
1	0 (0)	1 (6)		
>2	1 (1)	1 (6)		
**Distal end**			91	0.06
0	4 (5)	1 (6)		
1	0 (0)	0 (0)		
2	0 (0)	0 (0)		
3	2 (3)	0 (0)		
4	1 (1)	0 (0)		
5	2 (3)	0 (0)		
6	0 (0)	0 (0)		
7	2 (3)	1 (6)		
8	0 (0)	0 (0)		
9	2 (3)	4 (24)		
10-11	65 (83)	11 (65)		
Intramural haematoma	2 (14)	2 (11)	111	0.27
Max aortic diameter	5.6±1.1	5±1	35	0.24
Cerebral malperfusion	11 (14)	5 (33)	92	0.16
Renovisceral malperfusion	23 (26)	5 (25)	117	1
Extremity malperfusion	16 (26)	5 (38)	74	0.58

Supplementary Table S2: Univariate logistic regression model.

	OR	95% CI	p-value
**Neurological deficits**	7.45	1.69; 52.01	0.016*
**Spinal ischaemia**	1.04	0.22; 3.7	0.952
**Cerebral ischaemia**	3	0.81; 10.3	0.085
**Cerebral perfusion**	1.26	0.4; 3.68	0.681
**Oral anticoagulation**	3.79	0.89; 14.84	0.057
**Hypertension**	6.16	1.65; 40.13	0.019*
**Acute limb ischaemia, postoperative**	5.53	0.96; 32.14	0.046*
**Sepsis**	2.14	0.76; 5.85	0.138
**ARDS**	4.68	1.09; 18.28	0.028*
**Acute kidney injury, postoperative**	8.52	2.85; 31.67	<0.001*
**Reperfusion time (30min)**	1.33	0.84; 2.11	0.227
**Aortic cross clamp time (30min)**	1.19	0.88; 1.61	0.251
**Circulatory arrest time (30min)**	1.68	0.86; 3.28	0.125
**Total bypass time (30min)**	1.18	0.98; 1.41	0.074

OR: odds ratio; ARDS: acute respiratory distress syndrome; CI: confidence interval.

Supplementary Table S3: Summary of logistic regression coefficients after best subset selection.

	Estimate	Std. Error	Z value	p-value	OR	95% CI
**(Intercept)**	-3.614	0.734	-4.922	<0.001		
**Neurological deficits**	1.757	0.854	2.057	0.04*	5.79	1.22;41.6
**ARDS**	1.175	0.95	1.238	0.216	3.24	3.96;19.20

ARDS: acute respiratory distress syndrome; CI: confidence interval.
